# Beyond Inflammation: The Psychiatric Dimension of Familial Mediterranean Fever

**DOI:** 10.1192/j.eurpsy.2026.10451

**Published:** 2026-07-31

**Authors:** N. Bouayed Abdelmoula, E. Kazdaghli lagha, A. Kaabi, S. Kotti, B. Abdelmoula

**Affiliations:** LR23ES07 Genomics of Signalopathies at the service of Precision Medicine, Medical University of Sfax, Sfax, Tunisia

## Abstract

**Introduction:**

Familial Mediterranean fever (FMF) is a hereditary auto-inflammatory disease that commonly presents with recurrent febrile episodes and intense abdominal pain imitating surgical emergencies. Psychiatric comorbidities can complicate the clinical picture, delaying the diagnosis.

**Objectives:**

To highlight the diagnostic complexity of FMF in a patient with psychiatric symptoms and recurrent abdominal crises, and to emphasize the role of genetic testing in differentiating psychosomatic presentations of hereditary inflammatory diseases.

**Methods:**

A detailed clinical, biological and genetic evaluation was performed on a middle-aged patient with a long-standing history of unexplained febrile abdominal episodes and newly developed psychiatric symptoms. Genetic counseling and psychiatric evaluation have been integrated into the diagnostic pathway.

**Results:**

A middle-aged patient presented to our genetic counseling with a decades-long history of recurrent episodes of severe abdominal pain resembling an acute abdomen, often leading to unnecessary surgical evaluations. Initial symptoms included high fever and episodes of peritonitis-like pain that warranted surgery. Over time, the patient also developed psychiatric symptoms, including depression and suicidal ideation. A thorough examination excluded infectious or neoplastic causes. Laboratory tests during the episodes revealed elevated inflammatory markers. Genetic tests for FMF-related mutations have been conclusive, leading to the discovery of a familial homozygous point mutation in exon 10 of the MEFV gene. Psychiatric symptoms were managed during informed genetic counseling and psychiatric clinical care. This case illustrates the diagnostic challenge posed by FMF when the initial symptoms mimic surgical emergencies, aggravated by psychiatric comorbidities masking the central signs. Multidisciplinary collaboration was essential to achieve the diagnosis and optimize management.

**Conclusions:**

Clinicians should take into account psychiatric concerns in patients with hereditary periodic fever. Early recognition, genetic diagnosis and integrated medical-psychiatric care improve the outcomes of patients and their families.

**Disclosure of Interest:**

None Declared

